# Effective Immunotherapy in Bone Marrow Metastatic Melanoma Presenting with Disseminated Intravascular Coagulopathy

**DOI:** 10.1155/2018/4520294

**Published:** 2018-02-12

**Authors:** Bolanle Gbadamosi, Daniel Ezekwudo, Bhadresh Nayak, Zhou Yu, Sandra Gjorgova-Gjeorgjievski, Ming Xie, Colvin Robert, Ishmael Jaiyesimi, Marianne Huben

**Affiliations:** ^1^Beaumont Hospital, Oakland University School of Medicine, Department of Hematology and Oncology, Royal Oak, MI, USA; ^2^Beaumont Hospital, Oakland University School of Medicine, Department of Anatomic and Hematopathology, Royal Oak, MI, USA; ^3^Beaumont Hospital, Oakland University School of Medicine, Department of Diagnostic Radiology and Molecular Imaging, Royal Oak, MI, USA

## Abstract

Malignant melanoma is responsible for the majority of skin cancer deaths and is increasing in prevalence. Bone marrow (BM) involvement by melanoma is rare in the absence of widespread visceral disease. Here, we report the case of a 30-year-old female who presented to the hospital with back pain, low-grade fever, and easy bruising. She was found to be bicytopenic and in disseminated intravascular coagulopathy (DIC). Surprisingly, BM biopsy showed extensive involvement by metastatic malignant melanoma in the absence of visceral or brain metastasis. The unique presentation of this case and the challenge of management of a potentially treatable cancer in a critically ill patient are discussed, alongside a review of published cases of metastatic melanoma in the BM and an exploration of currently available treatment options. The excellent response of our patient to combined immune checkpoint inhibitors has yet to be paralleled in the available literature.

## 1. Introduction

Malignant melanoma represents about one percent of all skin cancers, but it is responsible for the majority of all skin cancer death. The average age of diagnosis of stage IV melanoma is 63 years, but there have been reports in young adults, especially females, aged 20 to 39 years. Historically, the 5-year survival rate of patients with stage IV metastatic melanoma is 15% to 20% [1]. Metastatic melanoma usually involves draining lymph nodes and adjacent skin first but eventually metastasizes to distant visceral sites. The lung is most commonly involved followed by the brain, liver, bone, and intestine [2]. Despite its predilection for distant metastasis, BM involvement by melanoma is rare in the absence of widespread disease. We present a case of malignant melanoma incidentally diagnosed after a BM biopsy for bicytopenia and DIC in the absence of widespread visceral metastasis, together with a systematic review of the literature and reported cases with metastatic melanoma in the BM.

## 2. Case Presentation

**Figure 1:**
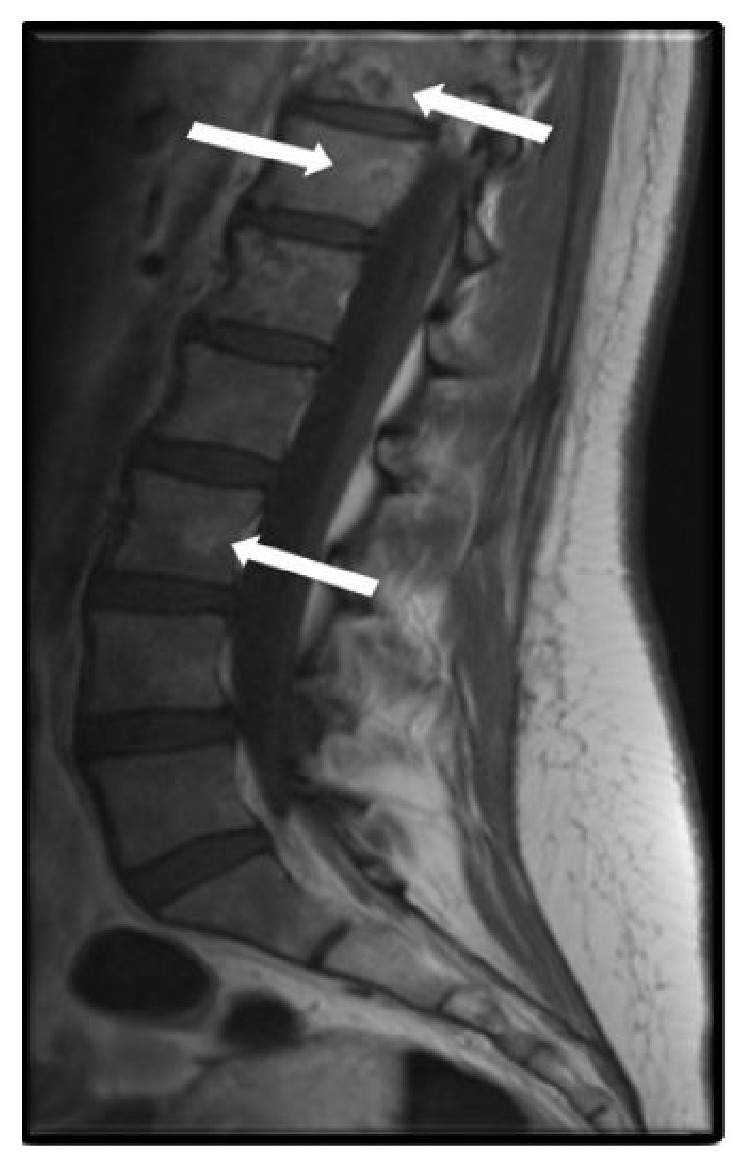
Sagittal T1 postcontrast image of the lumbar spine with multiple rim-enhancing lesions as indicated by white arrows.

A 30-year-old healthy female with no past medical history presented to the emergency room with worsening fatigue, easy bruising, low back pain, and low-grade fever. Physical examination revealed normal vital signs, mild oozing of blood from the gum, and a 3 cm right flank ecchymosis. Initial laboratory results showed severe thrombocytopenia, anemia, and coagulopathy (Table 1). Magnetic resonance imaging (MRI) of the thoracolumbar spine to evaluate low back pain showed pathologic process involving the marrow of all visualized osseous structures (Figure 1) which prompted a BM biopsy. Our differential diagnosis at the time included acute leukemia, lymphoma, aplastic anemia, myelophthisic process from metastatic solid cancer, or a rare infection.

**Figure 2:**
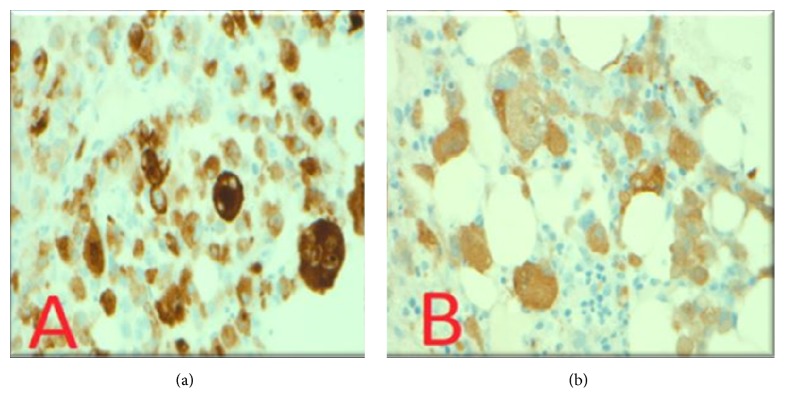
Immunohistochemical stain of bone marrow core biopsy with extensive involvement by poorly differentiated high-grade malignant neoplasm. Positive MART1 stain (a) and positive S100 stain (b).

**Figure 3:**
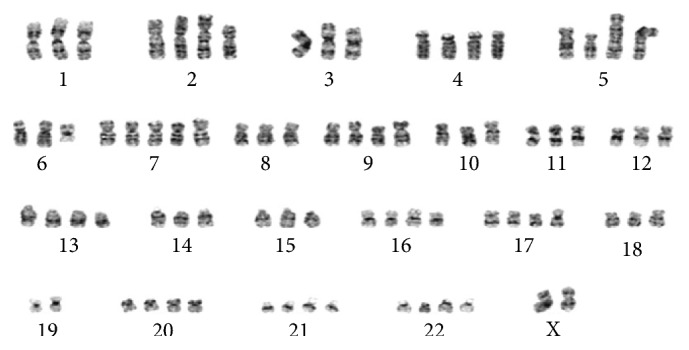
Cytogenetic analysis revealed a hypertriploid karyotype with multiple numerical and structural chromosome abnormalities. This cell demonstrates many, but not all, of the abnormalities designated in the composite karyotype, which was based on examination of twenty metaphase cells. Such complex karyotypes are common in high-grade malignancies, including metastatic melanoma.

BM aspirate flow cytometry showed no immunophenotypic evidence of acute leukemia, lymphoma, or plasma cell neoplasm. The BM core was hypercellular with 80% to 90% cellularity and normal blast percentage. Hematoxylin and eosin staining revealed 60% involvement by a poorly differentiated high-grade neoplasm with large pleomorphic nuclei, mitosis, prominent nucleoli, and abundant vacuolated cytoplasm; occasional multinucleated dark brown pigmented cells were also seen. The tumor cells were S100 and MART1 positive (Figure 2) but negative for CK7, TTF1, AE1/3, HepPar1, Glypican3, PAX8, HCG, CK5/6, P63, and OCT3/4, thus excluding other solid malignancy. Conventional cytogenetic analysis of the bone marrow biopsy revealed an abnormal complex hypertriploid karyotype with 77–79 chromosomes in 18/20 metaphase cells. The composite karyotype was designated as follows: 77–79〈3n〉XX, −X, add(2)(p21), +add(2)(q11.2), +4, add(5)(q11.2), +add(5)(q11.2), add(6)(q12), +7, +7, +8, +8, +9, −10, −11, +13, +15, +16, −19, +20, +21, +22[cp18]/46, XX (2) (Figure 3). Metastatic melanomas often present complex polyploid karyotypes such as the one identified in this study [3]. Molecular studies showed the tumor positive for BRAF V600E and negative for NRAS and c-kit.

**Figure 4:**
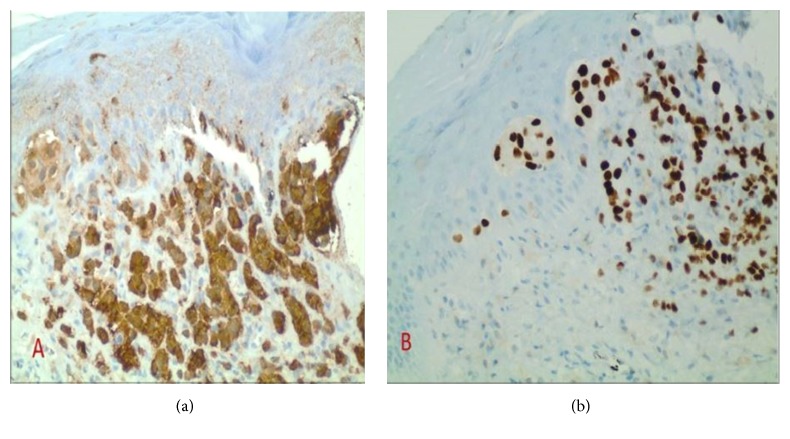
Immunohistochemical stain of the scalp lesion. Positive S-100 (a) 20x, SOX-10 (b) 20x. S-100 is a cytoplasmic stain, proving the malignant cells are originating from the neural crest derived tissue (melanocytes, glial cells, and Schwann cells). SOX-10 is a nuclear stain, confirming that the malignant cells are melanocytes.

**Figure 5:**
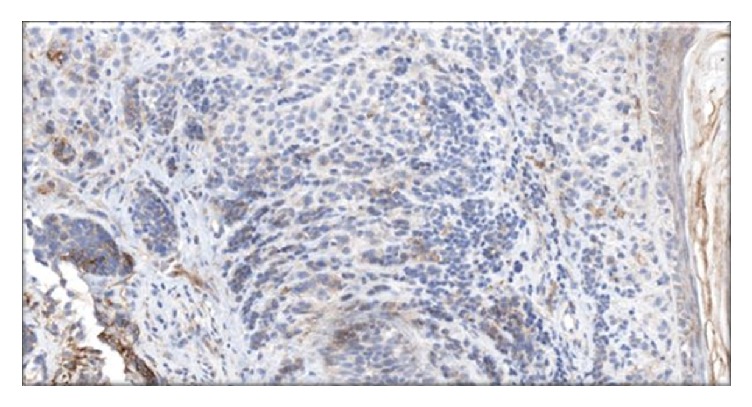
FDA-approved immunohistochemical stain for PD-L1, clone 28-8, (OPDIVO®) positive in 10.0% tumor cells with weak intensity.

A final diagnosis was made of metastatic melanoma of the BM presenting with DIC. A complete metastatic workup with CT of the chest, abdomen, and pelvis, as well brain MRI, showed predominantly osseous and BM metastatic disease with no other visceral involvement. A full dermatologic and ophthalmology examination showed a suspicious scalp lesion. Biopsy of the scalp lesion (Figure 4) revealed prominent intraepidermal atypical melanocytes with pagetoid spread, as well as the presence of well-formed junctional melanocytic nests, characteristic for malignant melanoma with superficial spreading pattern and stained positive for S-100 and SOX-10. The lesion was at least 2 mm deep (extending to the deep biopsy edges), Clark level IV with a mitotic rate of 2/mm^2^, and possible partial regression. Lymph-vascular invasion, perineural invasion, or ulceration was not identified. Tumor cells had 10% PD-L1 expression on formalin-fixed-paraffin embedded tissue sections using the United States Food and Drug Administration (FDA) approved programmed death ligand 1 (PD-L1) Immunohistochemical (IHC) stain 28-8 pharmDx, a monoclonal rabbit anti-PD-L1 (Figure 5). The patient required a 10-day intensive care unit (ICU) admission for management of DIC that was complicated by multiple bleeding episodes. She received a total of 21 units of pooled cryoprecipitate, 15 units of apheresed platelet, 13 units of packed red blood cells, and 8 units of fresh frozen plasma.

**Figure 6:**
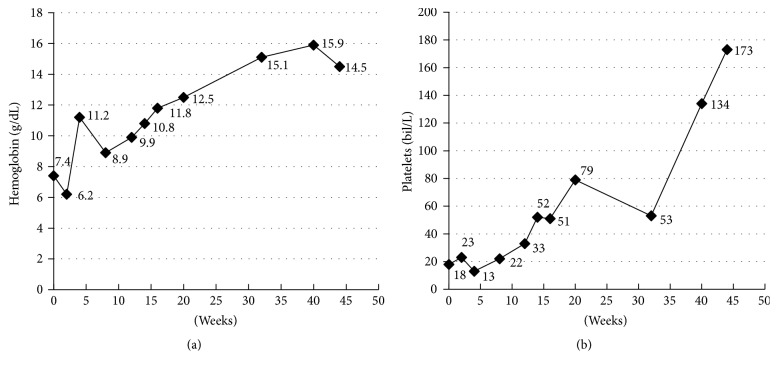
Trend of hemoglobin (a) and platelet (b) lab values.* Week 0*: at diagnosis. Treatment was initiated on* Day 3* and patient was transfusion-independent by* Week 8*.

Systemic therapy with nivolumab, a programmed death 1 (PD-1) inhibitor, at a dose of 240 mg every 2 weeks, and ipilimumab, a cytotoxic T-lymphocyte-associated antigen 4 (CTLA-4) inhibitor at a dose of 3 mg/kg every 3 weeks for 4 cycles, was started immediately after diagnosis. The therapy choice was based on the results of the Checkmate 067 trial [4] that showed better objective response rate (ORR) in the nivolumab-plus-ipilimumab arm compared to nivolumab alone and even better than ipilimumab alone; ORR was 72.1% (95% CI, 59.9 to 82.3) in the nivolumab-plus-ipilimumab group, 57.5% (95% CI, 45.9 to 68.5) in the nivolumab group, and 21.3% (95% CI, 12.7 to 32.3) in the ipilimumab group. Robert et al. reported ORR of 32.9% for pembrolizumab in KEYNOTE 002. The patient had an excellent response to the combined immune-checkpoint inhibitors, becoming transfusion-independent after 2 treatment cycles followed by complete resolution of bicytopenia (Figure 6).

Despite anticipated toxicity with this combination regimen, the patient has had no adverse effects to date. As of this report, she has received a total of 20 doses of nivolumab and 4 doses of ipilimumab, also consistent with results in the Checkmate 067 study, where the number of doses received in the nivolumab-plus-ipilimumab group ranged from 1 to 39 [4].

## 3. Discussion

An extensive literature search was conducted using PubMed, Scopus, Embase, Web of Science, and Google Scholar using search terms of “metastatic melanoma,” “bone marrow,” and “disseminated intravascular coagulopathy.” Twenty-nine cases of metastatic melanoma in the BM were found (Table 2). The median age at diagnosis was 55 (range: 5–81) years; the primary lesions were cutaneous in 15 (52%) cases, ocular in 5 (17%) cases, anal mucosa in 1 (3%) case, tonsil in 1 (3%) case, and pleural in 1 (3%) case, and 6 (21%) cases had an unknown primary lesion site. Presenting symptoms included fever, fatigue, bleeding, and back pain; only one previously published case presented with DIC as our patient did. Most of the patients were critically ill and only 5 (17%) received systemic chemotherapy. One (3%) patient was treated with pembrolizumab with good response, another one (3%) patient, a 5-year-old boy treated with ipilimumab, died 4 months after diagnosis, and one patient treated with nivolumab died 3 months after diagnosis. Overall, death within 6 months of diagnosis was reported in 15 (52%) patients, 1 (3%) patient had a good response, and the outcome was unknown for 13 (45%) patients. Of the cases reviewed, none were treated with combination immune-checkpoint inhibitors, as was the course of treatment for our patient.

The pattern of metastasis in melanoma usually initially involves draining lymph nodes and adjacent skin then, distant sites with the lung being the most common, followed by the brain, liver, bone, and intestine [2]. Adult solid cancers with high propensity for BM metastasis include breast, lung, gastric, prostate, and Ewing sarcoma [32]. Metastasis of malignant melanoma to the BM is rare, especially at the time of diagnosis, and approximately 5% to 7% of BM metastatic melanoma occurs with widespread tumor dissemination [33]. A proportion of BM metastasis of malignant melanoma occurs in the absence of an identifiable primary tumor, in situations where it is generally believed that the primary tumor has regressed [34].

Melanoma generally demonstrates unique, well-characterized receptor-ligand interactions during metastasis, as evidenced by the liver being the most common site of systemic metastasis of uveal melanoma; cutaneous melanoma is the most common cancer to metastasize to the submucosa of the small intestine. Also, expressions of integrin Alpha-v beta-3 (avb3), integrin *α*4*β*1, and p75 Nerve Growth Factor Receptor (NGF-R) by melanoma cells have been correlated with a tendency for lung, lymph node, or brain metastases, respectively [35–37]. There is no established receptor-ligand relationship associated with malignant melanoma metastasizing to the BM. The mechanism of BM metastasis of malignant melanoma can be linked to the concept of premetastatic niche (PMN), where Vascular Endothelial Growth Factor Receptor 1- (VEGFR1-) positive BM-derived hematopoietic stem cells are mobilized by factors secreted from the melanoma, to form a PMN with a tumor-receptive environment in the BM prior to the arrival of metastatic tumor cells. These PMN cells thus dictate the metastatic pattern, directing melanoma cells to the BM [38].

Therapeutic options for metastatic melanoma have changed tremendously in the last 5 years following the approval of immune checkpoint inhibitors and targeted therapies against BRAF mutations. Chemotherapy remained the first-line agent in the treatment of metastatic melanoma for about 4 decades [39–41].

The immunotherapy era for the treatment of metastatic melanoma started with approval by FDA in 1998 of interleukin-2 (IL-2), a cytokine that triggers production of lymphokine-activated killer (LAK) cells, which detect and cause the death of cancer cells. Trials reported CR rates of approximately 7%, and partial remission (PR) rates of 10% [42, 43], with the best response in patients having soft tissue and lung metastases. There was an associated durable response and survival benefit in a small group of patients; however, IL-2 use was limited by toxicity. Vaccine therapies have been investigated in basic and clinical research both in adjuvant and in metastatic settings. Initial results from phase II trials were promising; however, a phase III trial of CancerVax was prematurely stopped because of lack of efficacy [44–46].

In March 2011, ipilimumab, a human monoclonal antibody against the CTLA-4 receptor expressed on activated T-cells, was approved for treatment of metastatic melanoma. Treatment with ipilimumab showed a response rate (RR) of 5% to 15% across clinical trials and overall survival benefit, especially when combined with DTIC, though at the cost of toxicity [47]. Pembrolizumab, a humanized anti-PD-1 antibody, received FDA approval for advanced melanoma in 2014 based on clinical trial results with an outstanding RR of 38% and increased tolerability relative to other approved medications. Melanoma cells highly express PD-L1 as an adaptive mechanism for protection against the immune system. PD-1 receptors are expressed on the surface of CD8+T-cells and interact with corresponding ligands (PD-L1 and PD-L2) expressed by the tumor cells, resulting in inhibitory downstream signaling and cancer proliferation through the evasion of immune-mediated death [48, 49]. When compared with ipilimumab, pembrolizumab showed significantly better progression-free survival (PFS), OS, and less high-grade toxicity in treatment-naive advanced melanoma patients [50]. Studies with nivolumab, another anti PD-1, showed similar result.

Combination immunotherapy, as received by our patient, has been investigated in many clinical trials. Phase 1 trial of combination nivolumab and ipilimumab in newly diagnosed advanced melanoma showed clinical activity with ORR of 40% and deep tumor regression in a large proportion of patients, irrespective of absolute lymphocyte count or PDL-1 expression [51]. This result was reproduced in Checkmate 067, a 3-arm randomized phase III trial of ipilimumab alone, ipilimumab plus nivolumab, or nivolumab alone, where results showed statistically significantly longer PFS and OS with nivolumab plus ipilimumab or nivolumab alone than with ipilimumab alone [4, 52]. The phase II Keynote 024 and Anti-PD1 Brain Collaboration (ABC) trials of nivolumab and ipilimumab in combination also showed their effectiveness and safety in melanoma with brain metastasis. In second-line settings, ipilimumab alone had a better PFS compared to ipilimumab plus nivolumab, in patients with advanced melanoma after treatment failure on anti-PD-1 [53]. As expected, nivolumab led to a better objective response and fewer toxic effects than alternative chemotherapy in patients with advanced melanoma that progressed after ipilimumab or ipilimumab and a BRAF inhibitor [54]. Toxicity remains a major concern with combined immunotherapy as in our patient. The most common adverse events reported in Checkmate 067 in the nivolumab-plus-ipilimumab group were diarrhea (in 44.1% of patients), fatigue (in 35.1%), and pruritus (in 33.2%). One death due to neutropenia was reported in the nivolumab alone group, but none were reported in the nivolumab-plus-ipilimumab group. Cytopenia though uncommon could be an adverse effect of immunotherapy; our patient achieved a complete bone marrow response and normalization of CBC, and her cytopenia was due to marrow invasion by metastatic melanoma, thus inhibiting normal hematopoiesis. The rationale for BM response seen is likely related to the 10% PDL1 expression, known high mutation burden of malignant melanoma, and it also suggests high immunogenicity of the BM.

Approximately 40% to 50% of malignant melanomas express mutation in BRAF, a protein kinase that activates the MAP kinase/ERK-signaling pathway, causing cell growth and cancer replication [55]. In 2011, clinical trials showed 69% RR with vemurafenib, an anti-BRAF, though this response was short-lived in patients with BRAF-V600E mutation [56]. Dabrafenib, a reversible ATP-competitive inhibitor for BRAF, was also approved in March 2013. Combining inhibitors for mitogen-activated, extracellular signal-regulated kinase (MEK) and BRAF were associated with longer median PFS and OS [57]. The combination of anti-MEK and BRAF inhibitors is associated with delayed emergence of resistance and a reduced incidence of cutaneous hyperproliferative lesions.

Future directions in management of metastatic melanoma include vaccine, antiangiogenesis, and targeted therapies against NRAS, RAC1, ERK, and PI3K and c-KIT or PTEN. The role of percentage PDL-1 expression in the choice of immune checkpoint inhibitors, as well as the sequencing of therapy in patients with aberrant BRAF mutations, leaves intriguing questions to be answered.

## 4. Conclusion

Metastatic melanoma of the BM presenting with DIC in the absence of widespread metastasis is rare but remains a differential diagnosis in patients presenting with coagulopathy or cytopenia. Combination immunotherapy should be strongly considered in patients with good performance status, as our patient's excellent response suggests high immunogenicity of the BM, consistent with evolving data on immune checkpoint inhibitors in hematologic malignancies. To our knowledge, this is the first case report of BM metastatic melanoma in DIC treated with combination nivolumab plus ipilimumab. Our systematic review confirmed the dismal prognosis of this patient cohort with standard chemotherapy or treatment delay. Aggressive supportive care and prompt institution of immunotherapy can be lifesaving.

## Figures and Tables

**Table 1:** Laboratory result on admission.

Laboratory test	Patient	Reference range
WBC	11.1	4–10 × 10^9^/L
Hemoglobin	7.4	12–15 g/dL
MCV	90	80–100 fL
Platelet	18	150–400 × 10^9^/L
PT	29.3	9.3–12.4 seconds
INR	2.8	1–1.3
aPTT	44.0	23–30 seconds
D-dimer	>5000	0–499 ng/ml
Fibrinogen	<35	175–375 mg/dL
Schistocyte	1-2	<1/hpf
LDH	1719	100–238 U/L

WBC: white blood cells; MCV: mean corpuscular volume; PT: prothrombin time; INR: international normalizing ratio; aPTT: activated partial thromboplastin time; LDH: Lactate dehydrogenase.

**Table 2:** Reports of metastatic melanoma in the bone marrow, results of systematic review.

Authors	Age	Presenting symptoms	Primary	Treatment	Outcome
Battle and Stasney [5]	60	Weight loss	Ocular	Supportive	Death, 2 months
Rubinstein [6]	47	Cord compression	Occult	Thiouracil	Death, 2 months
Franklin et al. [7]	67	Back pain	Skin	Supportive	Death.
Franklin et al. [7]	18	Fatigue	Skin	Not reported	Unknown
Brown et al. [8]	66	Malaise, weight loss	Ocular	Supportive	Death, months
Basile et al. [9]	63	Abnormal CBC	Occult	Chemotherapy	Death, 6 months
Villarrubia et al. [10]	57	Petechiae	Skin	Not reported	Unknown
Invernizzi and Pecci [11]	34	Fever	Skin	Not reported	Unknown
Chim and Trendell Smith [12]	67	Red eye	Ocular	Not reported	Unknown
Basu et al. [13]	74	Fatigue	Anus	Not reported	Unknown
Basu et al. [13]	35	Cord compression	Tonsil	Not reported	Unknown
Batsis and Barry [14]	75	Altered mental status	Skin	Supportive	Death, 3 weeks
Uesawa et al. [15]	67	Back pain	Skin	Not reported	Unknown
Jain et al. [16]	22	Epistaxis	Occult	Chemotherapy	Unknown
Bhandari et al. [17]	62	Fever, joint pain	Ocular	Supportive	Unknown
Downing et al. [18]	49	Low back pain	Skin	Not reported	Unknown
Hsiao and Chen [19]	76	Low back pain	Ocular	Supportive	Death, 2 months
Suzuki et al. [20]	77	Bleeding, DIC	Occult	Supportive	Death, 1 week
Bertolotti et al. [21]	55	Dyspnea	Skin	Supportive	Death, days
Velasco-Rodríguez et al. [22]	75	Epistaxis	Skin	Chemotherapy	Death, 2 months
Serrier and Lesesve [23]	60	Dyspnea, back pain	Skin	Supportive	Death, days
Ferla et al. [24]	70	Fever, back pain	Skin	Supportive	Death, 3 months
Mirfazaelian et al. [25]	61	Abdominal pain	Occult	Not reported	Unknown
Kalodimos et al. [26]	81	Pancytopenia	Skin	Chemotherapy	Unknown
Volejnikova et al. [27]	5	Back pain, fever	Skin	Ipilimumab	Death, 4 months
Kassam and Shah [28]	79	Weight loss	Occult	Supportive	Death, 2 weeks
Fukumoto et al. [29]	26	Back pain	Skin	Nivolumab	Death, 3 months
Rosner et al. [30]	64	Fatigue, Fever	Skin	Pembrolizumab	Good response
Baniak et al. [31]	13	Pleural effusion	Pleural	Not reported	Unknown

## References

[1] Cancer Facts and Figures 2017. American Cancer Society. https://www.cancer.org/cancer/melanoma-skin-cancer/about/key-statistics.html

[2] De la Monte S. M., Moore G. W., Hutchins G. M. (1983). Patterned distribution of metastases from malignant melanoma in humans. *Cancer Research*.

[3] Nelson M. A., Radmacher M. D., Simon R. (2000). Chromosome abnormalities in malignant melanoma: Clinical significance of nonrandom chromosome abnormalities in 206 cases. *Cancer Genetics and Cytogenetics*.

[4] Larkin J., Hodi F. S., Wolchok J. D. (2015). Combined nivolumab and ipilimumab or monotherapy in untreated melanoma. *The New England Journal of Medicine*.

[5] Battle JD., Stasney J. (1941). Malignant melanoma cells in the bone marrow. *Archives of pathology*.

[6] Rubinstein M. A. (1949). Malignant melanoma diagnosed by marrow aspiration. *Acta Haematologica*.

[7] Franklin J. W., Zavala D. C., Radcliffe C. E. (1952). The detection of malignant melanoma by bone marrow aspiration; a report of two cases. *Blood*.

[8] Brown D., Boniuk M., Font R. L. (1990). Diffuse malignant melanoma of iris with metastases. *Survey of Ophthalmology*.

[9] Basile M., Moskowitz B., Harris J., Blumberg N., Bennett J. M. (1992). Malignant melanoma: Primary presentation in bone marrow and lymph node. *Medical and Pediatric Oncology*.

[10] Villarrubia J., de Misa R. F., Escribano L., Bellas C., Velasco J. L. (1995). Amelanotic Bone Marrow Infiltration Secondary to Pigmented Malignant Melanoma. *The Journal of Dermatology*.

[11] Invernizzi R., Pecci A. (2001). A case of metastatic malignant melanoma with bone marrow involvement. *Haematologica*.

[12] Chim C. S., Trendell Smith N. J. (2001). Primary malignant ocular melanoma: A bone marrow diagnosis. *British Journal of Haematology*.

[13] Basu D., Bhade B. A. (2002). Malignant melanoma metastatic to bone marrow. *Indian Journal of Pathology and Microbiology*.

[14] Batsis J. A., Barry M. J. (2006). Metastatic malignant melanoma presenting with hypercalcaemia and bone marrow involvement. *Journal of the European Academy of Dermatology and Venereology*.

[15] Uesawa M., Sato K., Ozaki K., Nagai T., Muroi K., Ozawa K. (2006). Bone marrow metastasis of malignant melanoma. *Internal Medicine*.

[16] Jain D., Singh T., Kumar N., Daga M. K. (2007). Metastatic malignant melanoma in bone marrow with occult primary site - A case report with review of literature. *Diagnostic Pathology*.

[17] Bhandari S., Jack F., Hussain K., Bell A. (2009). Metastatic melanoma in the marrow: A black and white diagnosis: Images in haematology. *British Journal of Haematology*.

[18] Downing K., Pitchford C., Royer M. (2012). Malignant melanoma presenting as lytic skeletal lesions and bone marrow infiltration. *The American Journal of Dermatopathology*.

[19] Hsiao S.-Y., Chen T.-Y. (2014). Uveal melanoma with diffuse bone marrow involvement. *Blood*.

[20] Suzuki T., Kusumoto S., Iida S., Tada T., Mori F. (2014). Amelanotic malignant melanoma of unknown primary origin metastasizing to the bone marrow: A case report and review of the literature. *Internal Medicine*.

[21] Bertolotti A., Conte H., Amazan E. (2013). Metastatic melanoma with leukaemia-like evolution. *Acta Dermato-Venereologica*.

[22] Velasco-Rodríguez D., Castellanos-González M., Alonso-Domínguez J. M., Martín-González M., Villarrubia J. (2013). Metastatic malignant melanoma detected on bone marrow aspiration. *British Journal of Haematology*.

[23] Serrier C., Lesesve J.-F. (2013). Metastatic malignant melanoma in the bone marrow. *Blood*.

[24] Ferla V., Freyrie A., Guidotti F., Bonoldi E., Gianelli U., Cortelezzi A. (2013). Bone marrow localisation of metastatic melanoma and synchronous leukaemic evolution of low-risk myelodysplastic syndrome. *Journal of Clinical Pathology*.

[25] Mirfazaelian H., Rezvani A., Daneshbod Y. (2013). Black bone marrow aspirate. *Journal of Postgraduate Medicine*.

[26] Kalodimos G., Skoufogiannis P., Lafioniatis S., Pappi V., Fanourgiakis P., Feritsean A.-M. (2016). Metastatic melanoma in bone marrow, report of a rare case. *Virchows Arch*.

[27] Volejnikova J., Bajciova V., Sulovska L. (2016). Bone marrow metastasis of malignant melanoma in childhood arising within a congenital melanocytic nevus. *Biomedical Papers*.

[28] Kassam S., Shah C. (2016). Amelanotic melanoma in the bone marrow. *Blood*.

[29] Fukumoto T., Sakaguchi M., Oka M., Nishimura M., Mukohara T., Nishigori C. (2017). Malignant melanoma with bone marrow involvement diagnosed from hypercalcemia: Development of a neural cell adhesion molecule stain. *The Journal of Dermatology*.

[30] Rosner S., Sen F., Postow M. (2017). Response after treatment with pembrolizumab in a patient with myelophthisis due to melanoma: the role of checkpoint inhibition in the bone. *Journal for ImmunoTherapy of Cancer*.

[31] Baniak N., Podberezin M., Kanthan S. C., Kanthan R. (2017). Primary pulmonary/pleural melanoma in a 13 year-old presenting as pleural effusion. *Pathology - Research and Practice*.

[32] Kilickap S., Erman M., Dincer M., Harputluoglu H., Yalcin S. (2007). Bone marrow metastasis of solid tumors: Clinicopathological evaluation of 73 cases. *Turkish Journal of Cancer*.

[33] Savage R. A., Lucas F. V., Hoffman G. C. (1983). Melanoma in marrow aspirates.. *American Journal of Clinical Pathology*.

[34] Baab G. H., McBride C. M. (1975). Malignant melanoma: the patient with an unknown site of primary origin. *JAMA Surgery*.

[35] Bakalian S., Marshall J.-C., Logan P. (2008). Molecular pathways mediating liver metastasis in patients with uveal melanoma. *Clinical Cancer Research*.

[36] Hieken T. J., Ronan S. G., Farolan M., Shilkaitis A. L., Das Gupta T. K. (1999). Molecular prognostic markers in intermediate-thickness cutaneous malignant melanoma. *Cancer*.

[37] Hieken T. J., Ronan S. G., Farolan M., Shilkaitis A. L., Das Gupta T. K. (1996). *β*1 integrin expression: A marker of lymphatic metastases in cutaneous malignant melanoma. *Anticancer Reseach*.

[38] Kaplan R. N., Rafii S., Lyden D. (2006). Preparing the “soil”: the premetastatic niche. *Cancer Research*.

[39] Huncharek M., Caubet J. F., McGarry R. (2001). Single-agent DTIC versus combination chemotherapy with or without immunotherapy in metastatic melanoma: A meta-analysis of 3273 patients from 20 randomized trials. *Melanoma Research*.

[40] Chapman P. B., Einhorn L. H., Meyers M. L. (1999). Phase III multicenter randomized trial of the Dartmouth regimen versus dacarbazine in patients with metastatic melanoma. *Journal of Clinical Oncology*.

[41] Middleton M. R., Grob J. J., Aaronson N. (2000). Randomized phase III study of temozolomide versus dacarbazine in the treatment of patients with advanced metastatic malignant melanoma. *Journal of Clinical Oncology*.

[42] Smith F. O., Downey S. G., Klapper J. A. (2008). Treatment of metastatic melanoma using Interleukin-2 alone or in conjunction with vaccines. *Clinical Cancer Research*.

[43] Atkins M. B., Lotze M. T., Dutcher J. P. (1999). High-dose recombinant interleukin 2 therapy for patients with metastatic melanoma: analysis of 270 patients treated between 1985 and 1993. *Journal of Clinical Oncology*.

[44] Morton D. L., Barth A. (1996). Vaccine therapy for malignant melanoma. *CA: A Cancer Journal for Clinicians*.

[45] Mitchell M. S., Harel W., Groshen S. (1992). Association of HLA phenotype with response to active specific immunotherapy of melanoma. *Journal of Clinical Oncology*.

[46] Mitchell M. S., Rechtman D. J. (1995). A randomized phase III trial of Melacine versus combination chemotherapy in patients with disseminated melanoma. *Canadian Journal of Infectious Diseases and Medical*.

[47] Robert C., Thomas L., Bondarenko I. (2011). Ipilimumab plus dacarbazine for previously untreated metastatic melanoma. *The New England Journal of Medicine*.

[48] Hamid O., Robert C., Daud A. (2013). Safety and tumor responses with lambrolizumab (anti-PD-1) in melanoma. *The New England Journal of Medicine*.

[49] Pardoll D. M. (2012). The blockade of immune checkpoints in cancer immunotherapy. *Nature Reviews Cancer*.

[50] Robert C., Schachter J., Long G. V. (2015). Pembrolizumab versus ipilimumab in advanced melanoma. *The New England Journal of Medicine*.

[51] Wolchok J. D., Kluger H., Callahan M. K. (2013). Nivolumab plus Ipilimumab in advanced melanoma. *The New England Journal of Medicine*.

[52] Wolchok J. D., Chiarion-Sileni V., Gonzalez R. (2017). Overall Survival with Combined Nivolumab and Ipilimumab in Advanced Melanoma. *The New England Journal of Medicine*.

[53] Zimmer L., Apuri S., Eroglu Z. (2017). Ipilimumab alone or in combination with nivolumab after progression on anti-PD-1 therapy in advanced melanoma. *European Journal of Cancer*.

[54] Weber J. S., D'Angelo S. P., Minor D. (2015). Nivolumab versus chemotherapy in patients with advanced melanoma who progressed after anti-CTLA-4 treatment (CheckMate 037): a randomised, controlled, open-label, phase 3 trial. *The Lancet Oncology*.

[55] Davies H., Bignell G. R., Cox C. (2002). Mutations of the BRAF gene in human cancer. *Nature*.

[56] Sosman J. A., Kim K. B., Schuchter L. (2012). Survival in braf V600–mutant advanced melanoma treated with vemurafenib. *The New England Journal of Medicine*.

[57] Long G. V., Stroyakovskiy D., Gogas H. (2015). Dabrafenib and trametinib versus dabrafenib and placebo for Val600 BRAF-mutant melanoma: A multicentre, double-blind, phase 3 randomised controlled trial. *The Lancet*.

